# Measurement and decomposition of education-related inequality in exclusive breastfeeding practice among Ethiopian mothers: applying Wagstaff decomposition analysis

**DOI:** 10.3389/fpubh.2024.1407210

**Published:** 2024-12-09

**Authors:** Anteneh Mengist Dessie, Melkamu Aderajew Zemene, Asaye Alamneh Gebeyehu, Denekew Tenaw Anley, Rahel Mulatie Anteneh, Natnael Moges, Ermias Sisay Chanie, Sintayehu Simie Tsega, Melaku Ashagrie Belete, Ermiyas Alemayehu, Natnael Kebede

**Affiliations:** ^1^Department of Public Health, College of Health Sciences, Debre Tabor University, Debre Tabor, Ethiopia; ^2^Department of Pediatrics and Child Health Nursing, College of Health Sciences, Debre Tabor University, Debre Tabor, Ethiopia; ^3^Department of Pediatric and Child Health, Debre Tabor University, Debre Tabor, Ethiopia; ^4^Department of Medical Nursing, School of Nursing, College of Medicine and Health Science, University of Gondar, Gondar, Ethiopia; ^5^Department of Medical Laboratory Sciences, College of Medicine and Health Sciences, Wollo University, Dessie, Ethiopia; ^6^Department of Health Promotion, School of Public Health, College of Medicine Health Sciences, Wollo University, Dessie, Ethiopia

**Keywords:** education, Ethiopia, exclusive breastfeeding, inequality, Wagstaff decomposition

## Abstract

**Background:**

Human breast milk, a naturally balanced source of infant nutrition, promotes optimal growth and health when exclusively fed for 6 months. Exclusive breastfeeding reduces common childhood infections, provides protection against some chronic illnesses, and contributes to achieving several Sustainable Development Goals. Despite its benefits, only 58% of Ethiopian women practice it, and the associated education-related inequality is not well documented. Thus, this study aims to quantify and decompose the education-related inequality in exclusive breastfeeding practice among Ethiopian mothers.

**Methods:**

A total of 1,504 weighted samples were studied using a Performance Monitoring for Action Ethiopia longitudinal panel survey dataset (2021–2023). Wagstaff normalized concentration index and its concentration curve were used to assess education-related inequality in exclusive breastfeeding practice. Wagstaff decomposition analysis was performed to decompose the concentration index and identify factors contributing to the observed education-related inequality. Significance was declared at *p*-value <0.05.

**Results:**

The overall prevalence of exclusive breastfeeding among Ethiopian women was 57.29% (95% CI: 54.79, 59.80%), with a greater concentration found among women with lower levels of education. This indicates an inequality that favors less educated women (pro-less educated inequality), as demonstrated by the Wagstaff normalized concentration index of −0.058 (95% CI: −0.113, −0.002). Factors that made a significant contribution to the observed education-related inequality in exclusive breastfeeding practice were residence (18.80%), region (29.31%), place of birth (−7.38%), and the wantedness status of the indexed pregnancy (82.58%). The indexed pregnancy’s wantedness was made a more elastic (elasticity = 0.282) contribution.

**Conclusion:**

The study identified a small yet significant education-related inequality in exclusive breastfeeding, favoring less educated women. Hence, emphasis should be placed not only on educating women but also on healthy habits that they can leave behind when they learn. Residence, region, place of birth, and indexed pregnancy’s wantedness significantly contributed to the observed education-related inequality. The elasticity value for each factor suggests that policy changes addressing these factors could readily reduce the observed inequality.

## 1 Introduction

Human breast milk is naturally balanced nutritious food for infants. Really, it is an ideal food for a child’s survival, growth, and development. Exclusive breastfeeding (EBF) is the practice of providing infants with only breast milk, including expressed breast milk, for the initial 6 months of life, with no introduction of additional liquids or solid foods, except for necessary medications (1). The World Health Organization (WHO) and United Nations Children’s Fund (UNICEF) recommend that all mothers should breastfeed their children exclusively for the first 6 months (2). For almost all infants, it is the simplest, most efficient, healthiest and cost-effective intervention to fulfill their requirements. However, only about 44% of infants aged 0–6 months worldwide were exclusively breastfed over the period of 2015–2020 (3).

Exclusive breastfeeding decreases the occurrence of common childhood illnesses such as diarrhea and acute respiratory infections. Moreover, it provides protection against some chronic illnesses and diseases, lowering the risk of asthma, obesity, type 1 diabetes, and sudden infant death syndrome (SIDS) in exclusively breastfed infants (4). Overall, it is estimated that EBF, combined with almost universal breastfeeding up to 1 year of age, has the potential to avert 13% of all deaths in children under the age of five in developing regions (5, 6). Beyond the direct health benefits for the child, exclusive breastfeeding plays a crucial role in achieving many of the Sustainable Development Goals (SDGs). These include SDG 2, addressing hunger and enhancing nutrition; SDG 3, reducing child mortality and mitigating the risk of non-communicable diseases; and SDG 4, promoting cognitive development and supporting education (7).

The Ethiopian government has implemented various interventions aimed at enhancing the practice of exclusive breastfeeding, including health information dissemination through various channels, the development of training materials and implementation guidelines for breastfeeding (8, 9), and the integration of breastfeeding into the primary healthcare system in alignment with the health extension program (10). Despite the recognized benefits and numerous efforts to promote exclusive breastfeeding, the practice remains below the recommended level. While breastfeeding is common in Ethiopia, with 97% of infants receiving breast milk at some point, only 58% of mothers exclusively breastfeed their infants (11). Additionally, the progress in exclusive breastfeeding has been minimal, with rates increasing only from 54.5% (95% CI: 49.9–59.0%) in 2000 to 59.9% (95% CI: 55.0–64.5%) in 2016 (12). Various socio-demographic, economic, and obstetric factors influence the exclusive breastfeeding practices of women in Ethiopia (11, 13–15).

Education is widely acknowledged as a key determinant of health outcomes, and its role in shaping exclusive breastfeeding practices has been explored in various contexts. Although highly educated women may have greater access to information about the benefits of exclusive breastfeeding, they often face challenges in practicing it due to the demands of their careers, which frequently require them to return to work soon after giving birth (13, 16). Additionally, concerns about body image among educated women (17) may further hinder their commitment to exclusive breastfeeding (18, 19). Therefore, this study aims to contribute to the existing literature by employing the Wagstaff decomposition analysis to measure and decompose education-related inequality in exclusive breastfeeding practices among Ethiopian women. This method allows for quantifying the extent of education-related inequality in exclusive breastfeeding practices, identifying the factors contributing to the observed inequality, and provides corresponding elasticities to elucidate how changes in the determinant relate to changes in the EBF practice.

## 2 Methods and materials

### 2.1 Study design, setting and period

A longitudinal panel survey design was employed to generate data regarding education-related inequality in exclusive breastfeeding practice among Ethiopian mothers. Ethiopia, where this study was conducted, is situated in the Horn of Africa (30–150N latitude and 330–480E longitude). The country occupies an area of 1.1 million square kilometers with an altitude that ranges from the highest peak at Ras Dashen (4,620 m above sea level) down to the Dallol depression, about 148 m below sea level. It has nine regional states (Afar, Amhara, Benishangul Gumuz, Gambela, Hareri, Oromia, Somali, Southern Nations Nationalities and People (SNNP), and Tigray) and two city administrations (Addis Ababa and Dire Dawa).

### 2.2 Data source, study populations, and sampling

This study utilized the performance monitoring for action Ethiopia 2021 (PMA-ET 2021) longitudinal panel survey datasets. The PMA Ethiopia collected cross-sectional data annually from 2019 to 2023 and longitudinal data (following pregnant women through 1 year postpartum) in two cohorts of women: cohort 1 (2019–2021) and cohort 2 (2021–2023). The second longitudinal panel survey that we utilized in this specific study was conducted between 2021 and 2023 in Oromia, Amhara, SNNP regional states, and one urban region, Addis Ababa, that collectively represent 85% of the population in Ethiopia.

The 2021 PMA Ethiopia panel survey employed two-stage stratified cluster sampling, where 162 enumeration areas (EAs) proportionally from both urban and rural strata and households (35 households per EA) were selected from the four regions at the first and second stages, respectively. Then, 2,298 pregnant women or less than 6 weeks postpartum in a given household were enrolled in the panel survey, and they were surveyed again after 6 weeks, 6 months, and a year postpartum. However, in this study, only 1,499 women (1,504 weighted samples) who participated in all three consecutive surveys (baseline, 6 weeks, and 6 months postpartum) and completed the interview were included in the final analysis. The 799 women who were dropped had a mean age of 26.93 years (SD = 6.65). Two hundred eighty-two (35.29%) of these women had identified as Orthodox, 280 (35.04%) as Protestant, 225 (28.16%) as Muslim, and 12 (1.52%) as belonging to other religions. They were from Amhara (144, 18.02%), Oromia (275, 34.42%), SNNP (271, 33.92%), and Addis Ababa (109, 13.64%). The majority, 91.61% (732), were married, with 41.93% (335) living in urban areas and 58.07% (464) in rural areas.

### 2.3 Variables of the study

The outcome variable for the study was exclusive breastfeeding which was coded as 0 for ‘not exclusively breastfeed’ and 1 for ‘exclusively breastfeed’. Women’s educational status was the measure of inequality in this study. Several sets of explanatory variables were used to explain the education-related inequalities in exclusive breastfeeding practice of women. Socio-demographic factors such as residence, wealth status, age, religion, marital status, and region, and pregnancy-related factors such as the wantedness status of the indexed pregnancy and place of delivery were all taken into account. The selection of these covariates was based on a review of the existing literature and their availability in our data source.

### 2.4 Statistical analysis

#### 2.4.1 Measuring education-related inequalities in EBF practice

All statistical analyses were conducted using Stata version 17. Two parameters were used to measure education-related inequality in EBF practice of women: (i) concentration curve (CC) and (ii) concentration index (C), using the highest level of school the women attended. The concentration curve plots the cumulative percentage of EBF practice (y-axis) against the cumulative percentage of women ranked by their educational status starting from the never attended school to the higher education (x-axis). There is an equality line (a 45°-line that extends from the lower left corner to the upper right corner) on the graph. A concentration curve above the line of equality indicates that EBF is disproportionately concentrated among women with low levels of education, and the reverse is true (20).

The concentration index, a common measure of inequality in health, was calculated to quantify the extent of education-related inequalities in the EBF practice of women (21). The C is twice the area between the concentration curve and the line of equality (21, 22). It can be written as follows (23):


$$C=2/n\mu \overset{n}{\underset{i=1}{\sum }}\mathrm{hiRi}-1$$

Where *h_i_* is the health outcome (EBF) for the *i*th woman; *μ* is the mean of *h*; *Ri* is the *i*th-ranked individual in the educational status distribution from the most disadvantaged (i.e., never attended school) to the least disadvantaged (i.e., higher education); and n is the number of women.

The concentration index value ranges from −1 to 1, where a negative value indicates that the EBF is disproportionately concentrated among women with low levels of education, and a positive value indicates that the EBF is disproportionately concentrated among women with high levels of education. If there is no inequality, it equals 0. However, when the outcome variable is a bounded variable (like EBF with values 0 and 1 in this study), the C will estimate the extent of inequality incorrectly. Thus, in this study, Wagstaff normalization was applied, and the Wagstaff normalized concentration index was calculated by dividing C by 1 minus the mean (1 – *μ*) (24). The statistical significance of the Wagstaff normalized concentration index was declared at a *p*-value <0.05.

#### 2.4.2 Decomposition of the concentration index

Wagstaff decomposition analysis was performed to decompose the concentration index and explain the contribution of each determinants on the observed overall education-related inequality in the EBF practice of women (22). This decomposition method was first introduced with a linear model. However, an appropriate statistical technique (probit model) that yields probabilities in the range 0–1 was applied to adjust it for non-linear health variables (25). Thus, the decomposition of the C in this study is based on regression analysis (maximum-likelihood probit model) of the relationship between an outcome variable and a set of determinants.

The overall concentration index comprises the explained part (the sum of the absolute contribution of all factors) and the unexplained part (the residual component). The final output of the Wagstaff decomposition process displays each factor’s coefficient, concentration index, its absolute and percentage contribution to the total observed education-related inequality, and the elasticity (the sensitivity of the outcome variable to that factor). Here, the regression coefficients reveal the relationship between the factor and the outcome variable (EBF), while the concentration index illustrates how the factor itself is distributed across the ranking variable (education). Elasticity is determined by weighting regression coefficients, giving higher weight to more prevalent determinants, and multiplying the result by the ratio of the mean of the determinant to the mean of the outcome. This elasticity provides insight into how changes in the determinant relate to changes in the outcome variable.

The absolute contribution, the contribution of factors to the overall inequality, is the product of the elasticity and concentration index of each factor. The percentage contribution (the adjusted one) is calculated by dividing each absolute contribution to the overall concentration index. Whereas, the unadjusted percentage contributions can be obtained by diving the absolute contribution to the total explained portion of the concentration index (i.e., the overall concentration index minus the residual). When the absolute contribution has a similar sign to the overall concentration index, the percentage contribution will be positive, indicating that the factor has that much of a positive contribution to the inequality. This suggests that the overall inequality in the outcome variable would decrease by that percentage if the variable was equally distributed to each level of educational status and had no effect on outcome variable. The interpretation will reverse when the absolute contribution has an opposite sign to the overall concentration index.

## 3 Results

### 3.1 Background characteristics of study participants

The median age of women was 26 years old with Inter Quartile Rang (IQR) of 22–31 years. Most of them were in the age group 25–34 years 744 (49.44%), married/in union 1,471 (97.81%), and from Oromia regional state 791 (52.58%). Regarding their educational status most of the women attended primary education 703 (46.71%) and only 121 (8.05%) women attended higher education. 1,107 (73.57%) women feel happy when they learned of pregnancy (Table 1).

**Table 1:** Background characteristics of study participants (*n* = 1,504).

Variables	Weighted frequency (%)	Exclusive breastfeeding status		*p*-value
		Yes	No	
Residence				
Urban	372 (24.77)	198	174	0.049
Rural	1,132 (75.23)	664	468	
Age of the women				
15–24	537 (35.70)	323	214	0.317
25–34	744 (49.44)	412	332	
≥ 35	223 (14.86)	127	96	
History of marriage				
Only once	1,326 (88.14)	758	568	0.814
Never and/or more than once	178 (11.86)	104	74	
Educational status of women				
Not attended school	468 (31.11)	274	194	0.043
Primary education	703 (46.71)	408	295	
Secondary education	212 (14.13)	126	86	
Higher education	121 (8.05)	54	67	
Partners educational status (*n* = 1,471)				
Not attended school	452 (28.48)	271	181	0.062
Primary education	624 (42.42)	349	275	
Secondary education	263 (17.88)	161	102	
Higher education	165 (11.22)	80	85	
Wealth status of the household				
Poor	594 (39.50)	347	247	0.489
Medium	295 (19.59)	175	120	
Rich	615 (40.91)	340	275	
Religion				
Protestant	426 (28.33)	200	226	<0.001
Orthodox	515 (34.22)	296	219	
Muslim	534 (35.49)	350	184	
Others	29 (1.97)	16	13	
Region				
Addis Ababa	70 (4.69)	34	36	<0.001
Amhara	329 (21.85)	203	126	
Oromia	791 (52.58)	492	299	
SNNP	314 (20.88)	133	181	
Feeling when learned of pregnancy				
Unhappy	252 (16.76)	133	119	0.377
Mixed happy & unhappy	145 (9.67)	87	58	
Happy	1,107 (73.57)	641	466	
Wantedness status of the indexed pregnancy				
Wanted then	953 (63.34)	536	417	0.423
Wanted later	468 (31.12)	272	196	
Not wanted at all	83 (5.54)	53	30	
Place of delivery				
Home	586 (38.99)	342	244	0.537
Health facility	918 (61.01)	519	399	

### 3.2 Prevalence of EBF

The overall prevalence of exclusive breastfeeding among Ethiopian women was 57.29 (95% CI: 54.79, 59.80%). The distribution of EBF was slightly higher among rural women: 198 (53.17%) and 664 (58.65%) of urban and rural women exclusively breastfeed their children, respectively.

### 3.3 Education-related inequality in EBF practice of women

**Figure 1:**
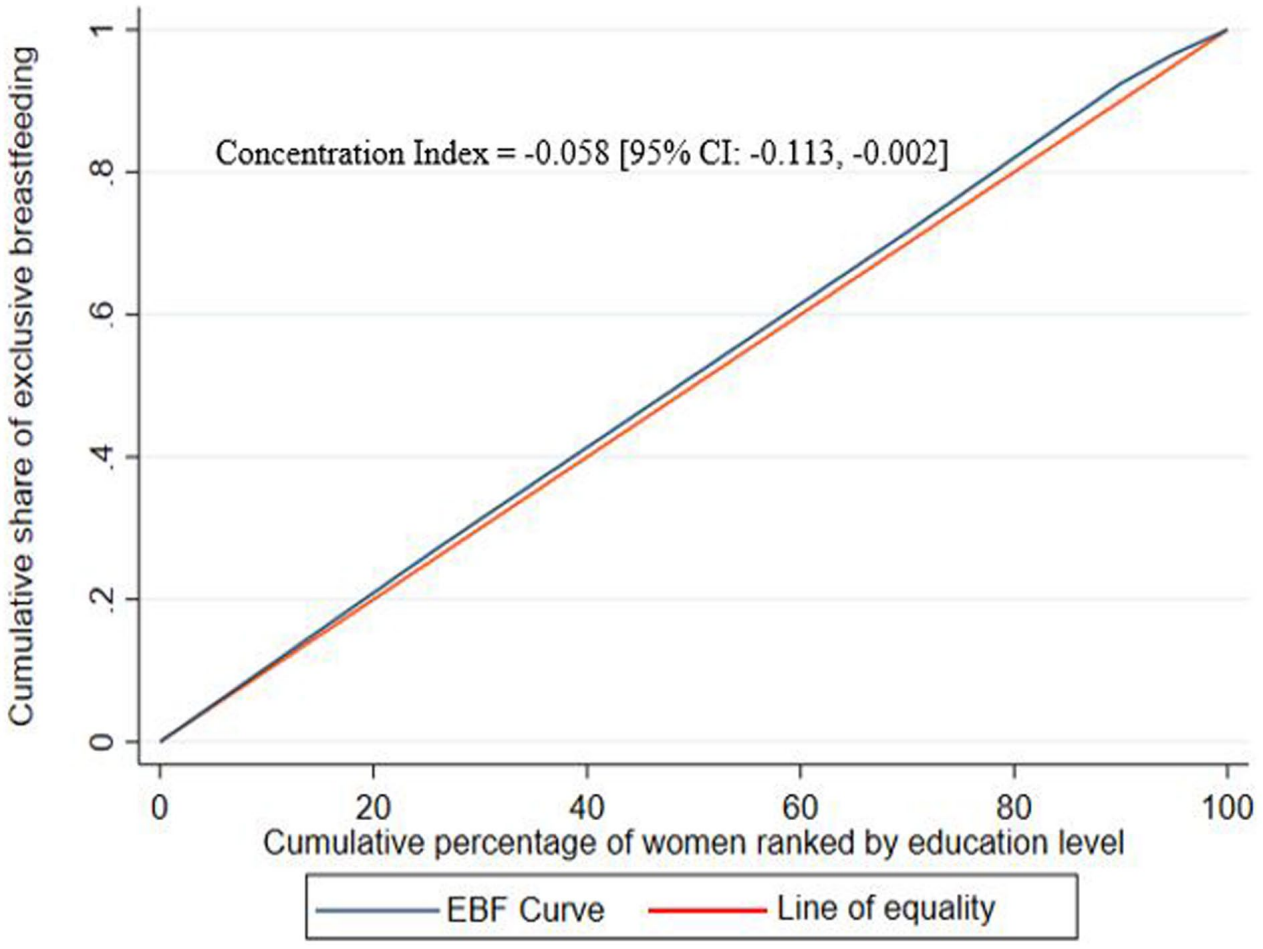
Concentration curve for the education-related inequality in exclusive breastfeeding practice among Ethiopian women.

The Wagstaff normalized concentration index for the education-related inequality in exclusive breastfeeding practice of women was −0.058 (95% CI: −0.113, −0.002, *p*-value = 0.039). Accordingly, there is a small but significant education gradient in exclusive breastfeeding practice. The negative sign reveals that EBF is disproportionately concentrated among the disadvantageous group (less educated women), meaning there is a pro-uneducated inequality in which women with low levels of education are more likely to practice EBF than the well-educated women. In order to get a distribution with an index value of zero (perfect equality), 4.35% (absolute value of the concentration index*75 (26)) of women who exclusively breastfeed would need to be redistributed from the less educated to the well-educated group of women. The concentration index is twice the area between the concentration curve and the line of equality (Figure 1).

### 3.4 Decomposition of education-related inequality in EBF

The Wagstaff decomposition analysis has been performed to decompose the overall education-related inequality in exclusive breastfeeding practices by variables and explain their contribution to the observed inequality. It quantifies how much of the observed education-related inequality was due to other variables. This was determined by calculating the coefficient, elasticity, concentration index, as well as the absolute and percent contribution for each factor. Given the pro-uneducated inequality observed in EBF practice in this study (indicated by a negative overall concentration index), if the independent variable is concentrated among the less educated group (negative concentration index) and exhibits a positive association with the outcome variable (positive coefficient), it would contribute positively (positive percentage contribution) to the overall inequality. Conversely, if the same variable shows a negative association with the outcome variable (negative coefficient), it would contribute negatively (negative percentage contribution) to the overall inequality. The reverse is true if the independent variable is concentrated among the well-educated group (positive concentration index).

Accordingly, rural women are more concentrated among the less educated group (concindex = −0.504) and being rural increases the probability of practicing exclusive breastfeeding (coefficient = 0.009). Consequently, 18.8% of the pro-uneducated inequality in EBF practice was attributed by residence differences, meaning if residence was equally distributed across the ranking variable and had no effect on the exclusive breastfeeding practice, the observed education-related inequality would decrease by 18.8 percent. The wantedness status of the indexed pregnancy contributes the most (82.58%) to the pro-uneducated education-related inequality in exclusive breastfeeding practices among women. Additionally, more than one-third (29.31%) of the inequality is explained by geographical location (region).

The act of giving birth at a health facility also explained −7.38% of the inequality, indicating that the total exclusive breastfeeding inequality would increase by 7.38% if place of delivery were equally distributed across the ranking variable and had no impact on exclusive breastfeeding practices. As indicated by the coefficient’s *p*-value, all other factors made a non-significant contribution to the overall concentration index.

In general, in this study, the combination of variables fitted in the model explained the majority (85.21%) of the education-related inequalities in exclusive breastfeeding practices among women. This implies that there is 14.79% an unexplained difference in exclusive breastfeeding practices that cannot be accounted by the factors studied. Most of the determinants exhibit a low level of elasticity, meaning that their contribution patterns remain stable and unaffected by potential policy changes. However, the wantedness status of the indexed pregnancy stands out as the determinant that shows a different pattern (bigger elasticity and contribution), suggesting that it could have a more responsive impact on changes in the EBF practice of women (Table 2).

**Table 2:** The Wagstaff decomposition analysis results showing the contribution of various explanatory variables to education-related inequality in EBF practice among Ethiopian women.

Variables	Coefficient (*p*-value)	Elasticity	Concentration index	Contribution	
				Absolute	Percentage
Residence					
Urban (**Ref.**)					
Rural	0.009* (0.045)	0.022	−0.504	−0.011	**18.80**
Age of the women					
15–24	0.032 (0.234)	0.043	0.114	0.005	−8.41
25–34	0.006 (0.352)	0.012	0.100	0.001	−2.07
≥35 (**Ref.**)					
Region					
Addis Ababa (**Ref.**)					
Amhara	0.105* (0.029)	0.095	−0.167	−0.016	**27.35**
Oromia	0.073* (0.032)	0.105	−0.011	−0.001	**1.96**
SNNP	−0.003 (0.113)	−0.004	−0.106	0.0004	−0.69
History of marriage					
Never or above once (**Ref.**)					
Only once	0.001 (0.456)	0.001	0.354	0.0005	−0.79
Religion					
Protestant (**Ref.**)					
Orthodox	0.070 (0.066)	0.114	0.133	0.015	−26.01
Muslim	0.052 (0.085)	0.072	−0.111	−0.008	13.79
Others	0.041 (0.723)	0.002	−0.325	−0.0007	1.15
Wealth status					
Poor	0.017 (0.691)	0.021	−0.482	−0.010	17.49
Medium	−0.020 (0.662)	−0.013	−0.255	0.003	−5.57
Rich (**Ref.**)					
Pace of delivery					
Home (**Ref.**)					
Health facility	0.003* (0.013)	0.009	0.477	0.004	**−7.38**
Wantedness status of the indexed pregnancy					
Wanted then	0.105* (0.003)	0.282	−0.170	−0.048	**82.58**
Wanted later	0.091 (0.150)	0.102	0.155	0.016	−26.99
Not wanted at all (**Ref.**)					
Total explained inequality				−0.0498	85.21%
Residual				−0.0082	14.79%
Overall inequality				−0.058	100%

Coefficient = Marginal effects from the probit model; * = significant at *p*-value < 0.05; Ref. = Reference category. The bold values indicate significance at a *p*-value < 0.05 for independent variables only.

## 4 Discussion

The aim of the present study was to examine the inequality in exclusive breastfeeding practices among women in Ethiopia based on their education levels, using nationally representative data. The overall prevalence of exclusive breastfeeding (57.29%) was found to be comparable to previous studies conducted in Ethiopia, where exclusive breastfeeding rates were reported as 57.3% (14), 58% (11), 50.1% (27), and 60.9% (15).

Our study revealed that exclusive breastfeeding is more prevalent among women with lower levels of education, indicating a pro-uneducated inequality. This disparity was quantified with a concentration index of −0.058, which is statistically significant at the 95% confidence level. This finding is consistent with previous evidence suggesting that EBF practices are often less favorable among more educated women (28–31). Several factors might explain this trend. Previous research has shown that women with higher education level are more likely to have demanding careers and shorter paid maternity leave. This professional pressure often necessitates returning to work before the infant reaches 6 months, thereby limiting the duration of exclusive breastfeeding (13, 16, 32, 33).

In addition, higher educational attainment is often associated with increased body image concerns, which can negatively impact a woman’s commitment to exclusive breastfeeding. Traditional perceptions that breastfeeding might alter a mother’s body shape and adversely affect her health can exacerbate these concerns, potentially discouraging educated women from adhering to EBF practices (17–19, 34). Another possible explanation is that highly educated mothers often have higher household incomes, enabling them to afford infant formula. This is particularly relevant in urban areas where formula feeding may be perceived as a sign of modernity or higher social status. Limited access to comprehensive breastfeeding education and the influence of marketing strategies promoting formula as a convenient alternative can further contribute to this preference, potentially leading to reduced rates of exclusive breastfeeding among more educated women (35, 36).

Contrastingly, other studies indicate that higher education levels often correlate with better knowledge of and adherence to breastfeeding guidelines. For instance, a global study reported that women with higher education levels were more likely to understand the benefits of EBF and thus were more likely to practice it (37). This is supported by the findings of similar study, which suggest that educated women have greater access to resources and support for breastfeeding (38). These studies argue that education equips women with the knowledge and skills necessary for successful breastfeeding, potentially leading to higher EBF rates. Therefore, improving EBF rates requires not only addressing educational disparities but also ensuring that all women receive comprehensive support to overcome barriers and promote successful breastfeeding practices.

In the Wagstaff decomposition analysis, factors such as residence, region, place of birth, and wantedness status of the indexed pregnancy had a significant contribution to the observed education-related inequality in exclusive breastfeeding practice among women in Ethiopia. Women residing in rural areas are predominantly found within the less educated group of women, and they are more likely to exclusively breastfeed their children. Consequently, residence makes a notable positive contribution to the observed pro-uneducated inequality in EBF, indicating that women with lower levels of education are more prone to engaging in exclusive breastfeeding. The finding is in line with other studies done in Ethiopia (39–41) and Malaysia (42). This could be explained by the fact that urban women will have more diverse employment opportunities, reducing the time they can spend with their infants and potentially affecting exclusive breastfeeding practices. Alternatively, it could be attributed to urban mothers having greater access to alternative infant feeding options compared to their rural counterparts.

Women from the Amhara and Oromia regions are disproportionately found in the less educated group of women and being from Amhara or Oromia is positively correlated with exclusive breastfeeding compared to women from Addis Ababa. Accordingly, approximately 29% of the education-related inequality in exclusive breastfeeding practices is attributable to this factor. Literature supports the assertion that women from larger cities, such as Addis Ababa, are less inclined to exclusively breastfeed their children for the recommended 6 months. A study from Addis Ababa indicated that only 44.2% of the mothers practiced EBF (43).

Conversely, women who give birth at a health facility are predominantly present within the well-educated group of women and are inclined to engage in exclusive breastfeeding. Thus, it has a negative percentage contribution (decrease the inequality by some extent) to the observed education-related inequality favoring less educated women in exclusive breastfeeding practices among women in Ethiopia. As a result, encouraging educated women to deliver at health facilities is crucial, as it enhances their likelihood of practicing exclusive breastfeeding (44), thereby further reducing the pro-uneducated education-related inequality in EBF practice.

The wantedness status of the indexed pregnancy made a significant, highly elastic, and big contribution to the observed pro-uneducated education-related inequality in exclusive breastfeeding practices of women in Ethiopia. Wanted pregnancies were mostly among women with lower levels of education, and these women were also more likely to breastfeed exclusively. It is supported by different studies (45, 46). Therefore, interventions should have to be designed to make all the pregnancies wanted among educated women to significantly reduce education-related inequality favoring less educated group in EBF practice of women.

There are, of course, still limitations that should be considered when interpreting the results. Firstly, the reliance on self-reported data, particularly concerning exclusive breastfeeding practices, raises the possibility of recall bias or social desirability bias, where participants might overreport or underreport their behaviors. Furthermore, the cross-sectional design of the study restricts the ability to establish causal relationships, as it only captures associations without confirming the directionality of those relationships. Unmeasured confounding factors that were not included in the analysis due to data limitations of the PMA survey may also influence exclusive breastfeeding practices, potentially confounding the results. For instance, factors such as maternal mental health, employment status/occupation, workplace support, cultural beliefs, and healthcare access and quality were not considered in this study, yet they can significantly impact breastfeeding behaviors.

## 5 Conclusion

The proportion of exclusive breastfeeding practice among women in Ethiopia was relatively low. Furthermore, exclusive breastfeeding practice was disproportionately concentrated on the less educated group of women (pro-uneducated concentration). Thus, emphasis should be placed not only on educating women, but also on healthy habits that they can leave behind when they learn. The government should also address reasons, such as having a job that offer only 120 working days of paid maternity leave, which may prevent mothers from abandoning their exclusive breastfeeding habits when they learn.

Residence, region, place of birth, and wantedness status of the indexed pregnancy made a significant contribution to the observed education-related inequality in exclusive breastfeeding practice among women in Ethiopia. The wantedness status of the indexed pregnancy made a more elastic contribution to the observed education-related inequality, implying that policy changes addressing pregnancy intention could readily reduce the observed inequality. Thus, the Ethiopian government is advised to design policies to ensure that every pregnancy is desired.

## Data Availability

Publicly available datasets were analyzed in this study. This data can be found at: https://www.pmadata.org/data.

## References

[1] WHO, UNICEF. Indicators for assessing infant and young child feeding practices country profiles. Geneva, Switzerland: WHO Press, World Health Organization (2010).

[2] WHO, UNICEF. Global strategy for infant and young child feeding World Health Organization (2003).

[3] WHO. Infant and young child feeding. (2023). Available at: https://www.who.int/news-room/fact-sheets/detail/infant-and-young-child-feeding (Accessed January 3, 2024).

[4] Clark SG Bungum TJ. Benefits of breastfeeding. Calif J Health Promot. (2003) 1:158–63. doi: 10.32398/cjhp.v1i3.527

[5] InfantCARE. Young child feeding practices: collecting and using data: a step-by-step guide: cooperative for assistance and relief everywhere Inc[CARE] (2010).

[6] Jones G Steketee RW Black RE Bhutta ZA Morris SS. How many child deaths can we prevent this year? Lancet. (2003) 362:65–71. doi: 10.1016/S0140-6736(03)13811-1 12853204

[7] Katsinde SM. Srinivas SC: breast feeding and the sustainable development agenda. Indian J Pharm Pract. (2016) 9:144–6. doi: 10.5530/ijopp.9.3.2

[8] Federal Ministry of Health FHDE. National strategy for infant and young child feeding Federal Ministry of Health, Family Health Department Ethiopia Addis Ababa (2004).

[9] Ethiopian Federal Ministry of Health U. Forces to promote safe breastfeeding Federal Ministry of Health Addis Ababa (2004).

[10] Fetene N Linnander E Fekadu B Alemu H Omer H Canavan M. The Ethiopian health extension program and variation in health systems performance: what matters? PLoS One. (2016) 11:e0156438. doi: 10.1371/journal.pone.0156438, PMID: 27227972 PMC4882046

[11] Muluneh MW. Determinants of exclusive breastfeeding practices among mothers in Ethiopia. PLoS One. (2023) 18:e0281576. doi: 10.1371/journal.pone.0281576, PMID: 36758057 PMC9910689

[12] Ahmed KY Page A Arora A Ogbo FA. Trends and determinants of early initiation of breastfeeding and exclusive breastfeeding in Ethiopia from 2000 to 2016. Int Breastfeed J. (2019) 14:1–14. doi: 10.1186/s13006-019-0234-9 31528197 PMC6740001

[13] Wake GE Mittiku YM. Prevalence of exclusive breastfeeding practice and its association with maternal employment in Ethiopia: a systematic review and meta-analysis. Int Breastfeed J. (2021) 16:1–14. doi: 10.1186/s13006-021-00432-x 34717673 PMC8557507

[14] Ayalew T. Exclusive breastfeeding practice and associated factors among first-time mothers in Bahir Dar city, North West Ethiopia: a community based cross sectional study. Heliyon. (2020) 6:e04732. doi: 10.1016/j.heliyon.2020.e04732, PMID: 32944666 PMC7481526

[15] Adugna B Tadele H Reta F Berhan Y. Determinants of exclusive breastfeeding in infants less than six months of age in Hawassa, an urban setting Ethiopia. Int Breastfeed J. (2017) 12:1–8. doi: 10.1186/s13006-017-0137-6 29142586 PMC5669024

[16] Neves PA Barros AJ Gatica-Domínguez G Vaz JS Baker P Lutter CK. Maternal education and equity in breastfeeding: trends and patterns in 81 low-and middle-income countries between 2000 and 2019. Int J Equity Health. (2021) 20:1–13. doi: 10.1186/s12939-020-01357-3 33413445 PMC7792102

[17] Lowe MA. Looking good: college women and body image, 1875–1930 JHU Press (2003).

[18] Brown A Rance J Warren L. Body image concerns during pregnancy are associated with a shorter breast feeding duration. Midwifery. (2015) 31:80–9. doi: 10.1016/j.midw.2014.06.003, PMID: 25151278

[19] Bigman G Wilkinson AV Homedes N Pérez A. Body image dissatisfaction, obesity and their associations with breastfeeding in Mexican women, a cross-sectional study. Matern Child Health J. (2018) 22:1815–25. doi: 10.1007/s10995-018-2583-1, PMID: 30003520

[20] Wagstaff A O'Donnell O Van Doorslaer E Lindelow M. Analyzing health equity using household survey data: a guide to techniques and their implementation World Bank Publications (2007).

[21] Wagstaff A Paci P Van Doorslaer E. On the measurement of inequalities in health. Soc Sci Med. (1991) 33:545–57. doi: 10.1016/0277-9536(91)90212-U 1962226

[22] Wagstaff A Van Doorslaer E Watanabe N. On decomposing the causes of health sector inequalities with an application to malnutrition inequalities in Vietnam. J Econ. (2003) 112:207–23. doi: 10.1016/S0304-4076(02)00161-6

[23] Kakwani N Wagstaff A Van Doorslaer E. Socioeconomic inequalities in health: measurement, computation, and statistical inference. J Econ. (1997) 77:87–103. doi: 10.1016/S0304-4076(96)01807-6

[24] Wagstaff A. The bounds of the concentration index when the variable of interest is binary, with an application to immunization inequality. Health Econ. (2005) 14:429–32. doi: 10.1002/hec.953, PMID: 15495147

[25] Doorslaer EV Koolman X Jones AM. Explaining income-related inequalities in doctor utilisation in Europe. Health Econ. (2004) 13:629–47. doi: 10.1002/hec.919 15259043

[26] Koolman X Van Doorslaer E. On the interpretation of a concentration index of inequality. Health Econ. (2004) 13:649–56. doi: 10.1002/hec.884 15259044

[27] Tewabe T Mandesh A Gualu T Alem G Mekuria G Zeleke H. Exclusive breastfeeding practice and associated factors among mothers in Motta town, East Gojjam zone, Amhara regional state, Ethiopia, 2015: a cross-sectional study. Int Breastfeed J. (2016) 12:1–7. doi: 10.1186/s13006-017-0103-3 28261318 PMC5327553

[28] Zhao J Zhao Y Du M Binns CW Lee AH. Maternal education and breastfeeding practices in China: a systematic review and meta-analysis. Midwifery. (2017) 50:62–71. doi: 10.1016/j.midw.2017.03.011, PMID: 28390256

[29] Tang K Wang H Tan SH Xin T Qu X Tang T. Association between maternal education and breast feeding practices in China: a population-based cross-sectional study. BMJ Open. (2019) 9:e028485. doi: 10.1136/bmjopen-2018-028485, PMID: 31467048 PMC6720234

[30] Gessese GT Woldeamanuel BT Demie TG Diriba Biratu T Handebo S. Breastfeeding performance index and associated factors among children aged 0–6 months in Ethiopia: analysis of the 2019 Ethiopia Mini demographic and health survey. Front Nutr. (2022) 9:970737. doi: 10.3389/fnut.2022.970737, PMID: 36263306 PMC9574351

[31] Quigley MA Carson C. Breastfeeding in the 21st century. Lancet. (2016) 387:2087–8. doi: 10.1016/S0140-6736(16)30534-7 27301815

[32] Chai Y Nandi A Heymann J. Does extending the duration of legislated paid maternity leave improve breastfeeding practices? Evidence from 38 low-income and middle-income countries. BMJ Glob Health. (2018) 3:e001032. doi: 10.1136/bmjgh-2018-001032, PMID: 30364395 PMC6195155

[33] Mirkovic KR Perrine CG Scanlon KS. Paid maternity leave and breastfeeding outcomes. Birth. (2016) 43:233–9. doi: 10.1111/birt.12230 26991788

[34] Gao LL SWC C You L Li X. Experiences of postpartum depression among first-time mothers in mainland China. J Adv Nurs. (2010) 66:303–12. doi: 10.1111/j.1365-2648.2009.05169.x 20423413

[35] Hernández-Vásquez A Vargas-Fernández R. Socioeconomic determinants and inequalities in exclusive breastfeeding among children in Peru. Front Nutr. (2022) 9:1073838. doi: 10.3389/fnut.2022.1073838, PMID: 36590201 PMC9798284

[36] Kent G. Global infant formula: monitoring and regulating the impacts to protect human health. Int Breastfeed J. (2015) 10:1–12. doi: 10.1186/s13006-014-0020-7 25784954 PMC4362817

[37] Rollins NC Bhandari N Hajeebhoy N Horton S Lutter CK Martines JC. Why invest, and what it will take to improve breastfeeding practices? Lancet. (2016) 387:491–504. doi: 10.1016/S0140-6736(15)01044-2 26869576

[38] Edmond KM Zandoh C Quigley MA Amenga-Etego S Owusu-Agyei S Kirkwood BR. Delayed breastfeeding initiation increases risk of neonatal mortality. Pediatrics. (2006) 117:e380–6. doi: 10.1542/peds.2005-1496 16510618

[39] Asfaw MM Argaw MD Kefene ZK. Factors associated with exclusive breastfeeding practices in Debre Berhan District, Central Ethiopia: a cross sectional community based study. Int Breastfeed J. (2015) 10:1–9. doi: 10.1186/s13006-015-0049-2 26269708 PMC4534166

[40] Shitie A Tilahun A Olijira L. Exclusive breastfeeding practice and associated factors among mothers of infants age 6 to 12 months in Somali region of Ethiopia. Sci Rep. (2022) 12:19102. doi: 10.1038/s41598-022-22051-0, PMID: 36351951 PMC9646813

[41] Awoke S Mulatu B. Determinants of exclusive breastfeeding practice among mothers in Sheka zone, Southwest Ethiopia: a cross-sectional study. Public Health Pract. (2021) 2:100108. doi: 10.1016/j.puhip.2021.100108, PMID: 36101636 PMC9461297

[42] Tan KL. Factors associated with exclusive breastfeeding among infants under six months of age in peninsular Malaysia. Int Breastfeed J. (2011) 6:2–7. doi: 10.1186/1746-4358-6-2 21284889 PMC3039569

[43] Elyas L Mekasha A Admasie A Assefa E. Exclusive breastfeeding practice and associated factors among mothers attending private pediatric and child clinics, Addis Ababa, Ethiopia: a cross-sectional study. Int J Pediatr. (2017) 2017:1–9. doi: 10.1155/2017/8546192, PMID: 29333171 PMC5733181

[44] Alebel A Tesma C Temesgen B Ferede A Kibret GD. Exclusive breastfeeding practice in Ethiopia and its association with antenatal care and institutional delivery: a systematic review and meta-analysis. Int Breastfeed J. (2018) 13:1–12. doi: 10.1186/s13006-018-0173-x 30026786 PMC6048887

[45] Shapiro-Mendoza CK Selwyn BJ Smith DP Sanderson M. The impact of pregnancy intention on breastfeeding duration in Bolivia and Paraguay. Stud Fam Plan. (2007) 38:198–205. doi: 10.1111/j.1728-4465.2007.00131.x, PMID: 17933293

[46] Friedson MS Arthur MML Burger AP. The relationship of pregnancy intentions to breastfeeding duration: a new evaluation In: Health and health care concerns among women and racial and ethnic minorities: Emerald Publishing Limited (2017). 13–37.

